# How Is the COVID-19 Pandemic Affecting Our Sexualities? An Overview of the Current Media Narratives and Research Hypotheses

**DOI:** 10.1007/s10508-020-01790-z

**Published:** 2020-08-05

**Authors:** Nicola Döring

**Affiliations:** Institute of Media and Communication Science, Department of Economic Sciences and Media, Ilmenau University of Technology, Ehrenbergstraße 29, 98693 Ilmenau, Germany

Coronavirus disease 2019 (COVID-19) first broke out in December 2019 in Wuhan, China, and has spread rapidly worldwide since the beginning of 2020. This new infectious disease is associated with a variety of symptoms and, in severe cases, leads to organ failure and death. On March 11, 2020, the World Health Organization (WHO) (2020a) declared the COVID-19 outbreak a global pandemic. Since then, the primary goal has been to slow down the spread of the novel coronavirus (SARS-CoV-2), which is responsible for the disease and is easily transmitted by direct and contact transmission.

To this end, travel restrictions, curfews, and contact bans have been imposed in numerous countries around the world, and all nonessential public institutions have been closed (COVID-19 shutdown or lockdown). Most political, cultural, religious, and sporting events have been canceled or postponed. People are being asked to wash their hands regularly and wear protective masks, to keep a minimum distance of 1.5 meters away from other human beings and to stay at home if possible (i.e., social distancing and self-isolation). By Spring 2020, more than half of the world population was in lockdown (Sandford, 2020). The COVID-19 pandemic is causing one of the world’s largest economic crises and is affecting the well-being of individuals; some stress factors, such as domestic isolation, lack of movement and social contact, loss of jobs and economic problems, supply bottlenecks, limited health and psychosocial care, and fear of and confrontation with infection and death, characterize life worldwide during the pandemic, but with great differences depending on the respective geographical region, socioeconomic situation, and personal circumstances.

## Sexuality-Related Effects of the COVID-19 Pandemic

In addition to many other pandemic consequences, the effects of COVID-19 on human sexualities have been intensively discussed on both mass media (press, radio, and television) and social media (Facebook, YouTube, Instagram, and Twitter; Döring & Walter, 2020). For example, the *New York Times* ran “Coronavirus and Sex: Questions and Answers” (Gunter, 2020), *VICE Magazine* reported “How Sex Workers are Dealing with Coronavirus” (Pringels, 2020), and the new Facebook group “*LGBTI COVID*-*19 Response”* was founded. This is gratifying because issues of sexual and reproductive health and rights and sexual well-being should be taken seriously, even (and perhaps especially) in times of crisis, as they are closely related to overall health and quality of life. This is where media reports can have an educational and supportive effect. At the same time, it should be noted that the media discourse on “sex and the coronavirus” naturally follows the old rule of “sex sells” (Streitmatter, 2004): the topic attracts attention and increases click rates, especially when it is sensationalized. Therefore, the accuracy of media narratives on sexuality and the novel coronavirus should always be questioned (Döring & Walter, 2020).

Researchers have also begun to address the sexuality-related effects of the COVID-19 pandemic. Comments on different sexuality-related aspects have been published in scientific journals (e.g., Ait Addi, Benksim, & Cherkaoui, 2020; Alpalhão & Filipe, 2020; Grubbs, 2020; Hall et al., 2020; Hough, 2020; Hussein, 2020; Joseph Davey, Bekker, Coates, & Myer, 2020; Mestre-Bach, Blycker, & Potenza, 2020; Sun et al., 2020), as well as first empirical studies (e.g., Chen et al., 2020; Jacob et al., 2020; Li, Li, Xin, Wang, & Yang, 2020; Riley, Sully, Ahmed, & Biddlecom, 2020; Sanchez, Zlotorzynska, Rai, & Baral, 2020; Yuksel & Ozgor, 2020) and research reviews (e.g., Rasmussen, Smulian, Lednicky, Wen, & Jamieson, 2020). Scientific societies provide statements on their websites (e.g., ASHA, 2020; ISSM, 2020; ISSWSH, 2020).

The first large-scale online surveys are already in progress: The Kinsey Institute (Indiana University), for example, launched a three-wave longitudinal study on “Sex and Relationships in the Time of COVID-19” (https://kinseyinstitute.org/research/covid-relationships.php) in March 2020. An international network of trans* organizations and research institutions initiated a global survey on “Measuring the impact of the COVID-19 pandemic on trans health & trans health care” (https://transcarecovid-19.com/). The Global Network of Sex Work Projects (NSWP) launched an international survey on the perceptions of sex workers and sex work organizations of COVID-19 effects in April 2020 (https://www.nswp.org/news/nswp-launch-covid-19-impact-survey). Many more national surveys and clinical studies are in the field or in preparation. In Germany, for example, in cooperation with the German Institute for Sexuality Pedagogy, sex educators were interviewed about their working conditions during the pandemic (https://www.tu-ilmenau.de/en/media-psychology-and-media-design/research/corona-survey/).

This commentary aims to inspire researchers and clinicians to further explore the sexuality-related implications of the COVID-19 pandemic. To this end, an overview of the effects that have been much discussed in the media and in academic journals is presented. The sexuality-related effects of the COVID-19 pandemic are addressed in four thematic blocks: (1) partnered sex, (2) solo sex, (3) sexual and reproductive health issues in vulnerable groups, and (4) sexual and reproductive healthcare and sexual education.

## COVID-19 Effects on Partnered Sex

How is COVID-19 changing partnered sex? Four main media narratives or research hypotheses of change could be identified in public and academic discourse.

### More Relationship Sex and a Coronavirus Baby Boom

In domestic COVID-19 isolation, couples are less distracted, have more time for each other, and can indulge in love making more often during the day, which—planned or unplanned—will inevitably lead to a “coronavirus baby boom” in nine months. This is at least a description found in mass media contributions (Döring & Walter, 2020) and academic papers (Ait Addi et al., 2020) alike. The press often speak of “Love in the Time of Corona” (e.g., BBC, 2020) in allusion to the bestselling novel “Love in the Time of Cholera” (García Márquez, 1988) that tells the story of two lovers who, after a life-long separation, finally get together and use the cholera quarantine on a ship for undisturbed togetherness. The belief in the “coronavirus baby boom,” which symbolizes a positive future, is so strong that names are already being created on social media for the new generation: they should not be called “Millennials” but “Coronials” (see hashtag #Coronials on Twitter or Instagram).

The narrative of the coronavirus baby boom is reminiscent of the hypothesis of the “blackout baby boom”: here, too, one imagines that couples have fewer distractions in the event of a power failure and can thus have sex undisturbed. However, the so-called blackout baby boom, in which newspapers are always happy to report on the basis of anecdotal examples, has never been confirmed on the basis of systematic empirical data. It is now more likely to be classified as a sexual urban legend (Brunvand, 1993): not only did the birth rate 9 months after the exceptional situation of a historical power failure in the U.S. not rise, but, in fact, it fell (Izenman & Zabell, 1981; Menaker, 1970; Udry, 1970). For most people, a prolonged blackout is a stressful and frightening interruption of their daily routine and thus is not particularly suited for relaxed romanticism and eroticism.

Perhaps a power outage lasting several hours is not the right comparison scenario either. Instead, terrorist attacks and natural disasters that create terror and put people in an exceptional situation for weeks and months might be the better reference. In fact, the available data here point to positive fertility effects that are explained by different theories. For example, terror management theory predicts that existential threats lead people to recall traditional and family values and thus become more willing to marry and reproduce. Attachment theory predicts that, in times of threat, people move closer together with their significant others. Replacement theory assumes that, in existential crises, people—consciously or unconsciously—conceive more children to compensate for the loss of life. The fertility effects of collective threat situations are complex and depend on various influencing factors (Evans, Hu, & Zhao, 2010; Rodgers, St John, & Coleman, 2005). They would therefore also need to be worked out more precisely for the COVID-19 pandemic. An Italian online survey with a self-selection sample of 1486 women and men of fertile age in stable heterosexual relationships (Micelli et al., 2020) revealed both a pandemic-related increase in the desire of parenthood (e.g., due to need for positivity) and a decrease (e.g., due to economic difficulties). A Turkish study with a sample of 58 married women of fertile age (Yuksel & Ozgor, 2020) showed that during the COVID-19 pandemic, the frequency of sexual intercourse in marriage increased significantly (from an average of 1.9 times to 2.4 times per week) compared to 6–12 months before; at the same time, however, the proportion of women intending to become pregnant decreased (from 32.7 to 5.1%). Thus, regarding the predicted coronavirus baby boom, whether these are intended or unintended pregnancies must always be questioned (see section below on pandemic-related barriers to family planning and abortion).

It should also be noted that the more relationship sex plus baby boom narrative always assumes that there is no risk of SARS-CoV-2 transmission in cohabiting couples because neither partner is infected or because both are already infected or immune. In discordant couples, however, partnered sex is considered unsafe and should—similar to other types of physical contact—be strictly avoided, as emphasized by media, health authority, and scientific society publications (e.g., ISSM, 2020; ISSWSH, 2020).

### Less Relationship Sex and the Rise in Divorce Rates

The romantic-optimistic picture of the “quarantine honeymoon” including a “coronavirus baby boom” has not remained unchallenged. Relatively quickly, a completely unerotic picture of the domestic COVID-19 isolation was drawn: one heard of couples who were mainly bored and annoyed with each other. Moreover, in domestic isolation, otherwise hidden injustices, gender asymmetries, and family conflicts can become unexpectedly apparent, as the *Washington Post* points out: “The truth is, with babysitters, nannies, day cares, schools and, for some folks, their jobs, out of the mix, relationship inequities could be a lot more fraught” (Dvorak, 2020). According to media narratives, a kind of “camp frenzy” and permanent dispute with subsequent soaring separation and divorce rates can be expected (Döring & Walter, 2020). Factors contributing to relationship stress during the pandemic are limited living space, demanding home schooling and home office duties as well as job loss, preexisting conflicts, physical and mental diseases.

Literature reviews show that mental health deteriorated significantly after the economic crisis in 2008 (Parmar, Stavropoulou, & Ioannidis, 2016) and in the course of quarantine measures related to earlier pandemics (Brooks et al., 2020). An increase in anger, anxiety, post-traumatic stress symptoms, depression or even suicidal tendencies usually has a negative impact on relationships and sexual life and might foster sexual dysfunctions and sexual deviance. Some studies also show that divorce rates increase in the aftermath of natural disasters (Cohan & Cole, 2002).

There is thus a clear need for psychosocial care: counseling, therapy, and crisis intervention are needed for individuals, couples, and families whose domestic conflicts and sexual problems have intensified during the pandemic and who are in separation crises. In addition, a special burden situation arises for couples who are being forced to be separated for a long period of time during the COVID-19 pandemic (e.g., due to travel bans or because one person is living in a nursing home due to old age, which has issued a visiting ban).

The *Washington Times* asked the following question: “Will coronavirus intimacy lead to a baby boom? Or a divorce tsunami?” (Dvorak, 2020). However, presumably, it is not at all about playing one effect against the other but rather about finding the determinants that ensure that some couples become closer during the coronavirus crisis, while others grow apart. Correspondingly ambivalent effects are also known from less dramatic situations such as holidays: some couples fall in love anew on vacation, and others fight all the time and separate afterward.

It should also be borne in mind that the effect sizes of the postulated effects may be small and the changes only short-term in the general population. Many couples might stick to their established patterns of communication and sexuality or return to them in the wake of the crisis. It is therefore important not only to focus on problematic changes and support needs but also to keep an eye on the persistence and resilience of couples and identify the factors that foster their successful coping.

### Less Casual Sex

As the examples quoted so far show, changes in sexuality as a result of the COVID-19 pandemic are often discussed using heterosexual monogamous couple relationships as an example. A different narrative emerges when one looks at partnered sex, which does not take place with domestic spouses and life partners but within the framework of alternative relationship models (e.g., friendships with benefits and polyamorous relationship networks) and is practiced as so-called casual sex, for example, in the context of one-night stands, hook-ups, sex dates, affairs or commercial sexual services. These forms of sexual interactions, which are more often part of the lifestyles of adolescents, singles, and LGBTQ + -identifying people, among others, have been largely prohibited in the COVID-19 pandemic mitigation measures. This means that many adolescents and adults have had to adjust to long weeks and months without intimate physical contact with other people.

There has been much media coverage about the fact that “bad times for singles” are dawning or that their “libido must take a break.” Newspapers have quoted surveys of heterosexual and queer dating services, whose members stated that since the beginning of the coronavirus crisis, they have had much less sex or no sex at all and have renounced casual sex (Döring & Walter, 2020). A rapid online survey of 1051 men who have sex with men from the U.S. showed that half of them had less sex partners (Sanchez et al., 2020). A Chinese online survey with a convenience sample of 459 women and men aged 18–45 years revealed a decrease in sexual partners, sexual frequency, and sexual risk taking during the pandemic (Li et al., 2020). The Editor of the journal *Sexuality and Disability* is asking for data on how people with disabilities are doing during the pandemic when their scheduled sexual surrogate and massage sessions are canceled and they remain without any sensual physical contact for weeks and months (Hough, 2020).

In addition to a descriptive narrative describing the living conditions of singles who adhere to social and sexual contact bans, a prescriptive narrative can also be observed on social media. This narrative addresses how to encourage or even force people to stop having casual sex during the COVID-19 pandemic. In the newly formed Facebook group “LGBTI COVID-19 Response,” for example, one member suggested in a prescriptive less casual sex narrative that during the COVID-19 pandemic, it would be best to shut down Grindr, Scruff, GROWLr, and all other queer dating apps to stop casual sex. A heated discussion with dozens of posts followed.

Especially in LGBTQ + communities, comparisons between coronavirus narratives and HIV narratives are obvious. The establishment of safer sex, Treatment as Prevention (TasP), and, more recently, Pre-Exposure Prophylaxis (PrEP) significantly reduced the fear of infection in MSM with regard to HIV. Will the novel coronavirus, which is much easier to transmit than HIV, threaten the newfound freedom from fear and foster a new stigmatization of nonmonogamous lifestyles?

How, from a scientific point of view, is an appropriate risk assessment to be made? How do the risks of a weeks- or months-long lack of physical intimacy compare with the increased risk of SARS-CoV-2 infection through casual sex? Is there possibly a pragmatic middle ground of risk reduction, which lies between a risky “carry on as before” mentality on the one hand and total abstinence from partnered sex on the other? Anecdotally, there are reports of singles who limit their casual sex to a small circle of people to reduce the risk of SARS-CoV-2 transmission or who go into self-isolation together as a group and then only have sex among each other. This procedure is recommended on YouTube by an Australian doctor from the LGBTQ + community, among others (Forgan-Schmith, 2020), and discussed in the scientific literature (Turban, Keuroghlian, & Mayer, 2020).

Finally, the expected reduction in casual sex should reduce the transmission of HIV and STI, and first evidence of a corresponding reduction in new infections is already available in the literature (e.g., Alpalhão & Filipe, 2020; Junejo, Girometti, McOwan, Whitlock, & Dean Street Collaborative Group, 2020).

### More Telephone and Online Sex

The fact that partnered sex with people outside the household poses a significant risk of SARS-CoV-2 transmission is undisputed in view of its easy transmission through respiratory droplets and physical intimacy (Cipriano, Giacalone, & Ruberti, 2020). However, what remains to be considered is interpersonal sexual contact at a distance. These technology-mediated sexual interactions and relationships have been known since the 1990s under labels such as cybersex, virtual sex, electronic sex, chat sex, cam sex, or more recently, sexting (Courtice & Shaughnessy, 2017). In view of the coronavirus contact bans, such technology-mediated interpersonal sex contacts suddenly enjoy great popularity and are so much in the public eye that, in March 2020, a narrative of more telephone sex and more online sex was identified in German-speaking media as the most popular partnered sex media narrative (Döring & Walter, 2020). Similar to the narrative of less casual sex, the narrative of more phone sex and online sex exists with descriptive and prescriptive variants.

On the one hand, it is observed and described that an increasing number of people prefer to resort to telephone and online sex instead of face-to-face sex dates. On the other hand, this approach has been explicitly recommended. The New York City Health Department made headlines worldwide when it published tips on safe sex during the COVID-19 pandemic on March 21, 2020, positioning itself as being surprisingly inclusive and sex-positive for a state agency (NYC Health Department, 2020): “If you usually meet your sex partners online or make a living by having sex, consider taking a break from in-person dates. Video dates, sexting or chat rooms may be options for you.”

It is remarkable that online sex, including sexting, which is often frowned upon and even declared a pathological or deviant risky behavior (see Döring, 2014), has now officially advanced to a disease prevention behavior. Health organizations worldwide recommend telephone and online sex as alternatives to in-person sex dates. The International Society for the Study of Women’s Sexual Health (ISSWSH, 2020) postulates the following: “The new ‘really safe’ sex in many cases may require ‘e-sex’.”

Since the beginning of the COVID-19 pandemic, the media have been providing tips, tricks, and advice for good telephone and online sex. Such tips are quite useful, as phone and online sex require specific knowledge and skills. Therefore, in times of a pandemic, specific sexual education is in demand. The technical side of mediated sex requires knowledge and skills regarding the selection of the necessary equipment (microphone, camera, and light) and the appropriate platform or app (e.g., Zoom, Skype, Tinder, Grindr, etc.) as well as the correct handling (e.g., appealing camera angle and correct privacy settings). The sexual and social side of mediated sex requires knowledge and skills of how to express sexual desires and fantasies interactively and consensually with other people through text, pictures, audio, and video. This includes sexual interactions not only between two people but also between several people and in groups, as well as the integration of sexual scripts from kink, fetish, BDSM, and LGBTQ + contexts. In the meantime, queer sex parties are being organized online with “Instagram DJs” and “Skype darkrooms.” For example, the online edition of London’s “Naked Gay Party SBN,” which normally has approximately 600 guests offline, welcomed more than 3000 visitors in the video chat room on March 29, 2020 (Attitude, 2020).

In the course of the COVID-19 pandemic, a destigmatization and normalization of technology-mediated sexual interactions has been observed, which is desirable in principle, since these interactions have been part of the sexual repertoire of various populations for about a quarter of a century and are experienced positively by the majority (Döring, 2009; Döring & Mohseni, 2018). Conversely, it is problematic if the appreciation of the qualities of technology-mediated sex—especially its usefulness during face-to-face contact bans—immediately turns into glorification, as can be observed in some press articles (Döring & Walter, 2020). The sexual literacy to be promoted through sexual education in connection with technology-mediated sexual interactions should also include awareness of risks and protective measures (Alpalhão & Filipe, 2020; Turban et al., 2020). This concerns, for example, the prevention of and intervention in cases of online sexual harassment, online grooming, online stalking, revenge pornography, online dating scams, etc. Sexual education should consider these topics, and in counseling and therapy, one should be prepared to deal with conflicts and problems related to telephone and online sex and to support people in their search for satisfying technology-mediated sexual contact.

It would be interesting for sex researchers to investigate how technology-mediated sexual interactions are experienced and whether and how, for example, sexual scripts in online sex differ from sexual scripts in offline sex (cf. Döring, 2000). In the context of technology-mediated partnered sex research questions relating to remote-controlled sex toys that add haptic stimulation to the experience (Liberati, 2017) should also be considered, including new technologies that allow for kissing over distance (Cheok & Zhang, 2020). Pandemic-relevant research questions are also related to immersive virtual environments in which people experience sexual interactions mediated by avatars (Wardle, 2018).

Another relevant research question addresses who will switch from offline sex to online sex during the pandemic and who cannot or will not do so. Particular attention should be paid here, for example, to the situation of young people: for them, participation in online sex and sexting is partly illegal, depending on the national legal system and is generally considered very risky. At the same time, young people are often single and have a strong desire for sexual exploration and contact. What does “safer online sex” or “safer phone sex” look like for young people (Döring, 2014) under pandemic conditions?

Regarding senior citizens, contrary to the cliché of the “nonsexual elderly,” older people today also want to be sexually active more often. This raises the following question: Do single elderly people at home and in residential and care facilities have the possibility of finding suitable technology-mediated sexual expressions under the conditions of a pandemic? Are there sexual education offerings that support older people and people with disabilities in this and take their needs for sexual well-being seriously?

## COVID-19 Effects on Solo Sex

In addition to changes in partnered sex, changes in solo sex have also been intensively discussed, with four aspects of change particularly being suspected.

### More Masturbation

Those who spend more time in domestic isolation due to the COVID-19 pandemic also have more time and opportunity to masturbate. This at least is suspected by the public, and a corresponding descriptive narrative of more masturbation has been well visible in the media in Spring 2020 (Döring & Walter, 2020). This also applies to a prescriptive narrative of more masturbation, which directly calls on the population to masturbate more often. Masturbation is recommended because it reduces stress and anxiety, strengthens the immune system, fights boredom and frustration, and compensates for the lack of partnered sex, at least according to the arguments of the media (e.g., Die Bild, 2020), loosely referring to the state of research on the positive health outcomes of masturbation (e.g., Coleman, 2003; Levin, 2007; Robbins et al., 2011). The New York City Health Department (NYC Health Department, 2020) was quoted worldwide as stating the following: “*You* are your safest sex partner. Masturbation will not spread COVID-19, especially if you wash your hands (and any sex toys) with soap and water for at least 20 s before and after sex.”

The new enthusiasm with which the media and health authorities are celebrating masturbation may be a desirable step toward further destigmatizing and normalizing solo sex. At the same time, it is problematic when masturbation is officially recommended as an alternative to partnered sex. For some people masturbation might be a viable substitute, while others feel they cannot do without interpersonal intimacy.

Empirically, it is an open question whether, and if so, with whom, changes in masturbation frequency occur at all. In addition to pandemic factors that can have an increasing effect (e.g., more time at home), there are also pandemic factors that can have a dampening effect (e.g., fears and worries). It is also plausible that the majority of people will more or less stick to their preexisting masturbation habits.

### More Sex Toy Use

The most popular solo sex media narrative during the early phase of the COVID-19 pandemic has postulated the use of more sex toys (Döring & Walter, 2020). Toys are used for partnered sex, but even more often—especially with women—for solo sex (Döring & Poeschl, 2019), for which during coronavirus isolation, according to common belief, there is a particularly high opportunity.

The media narrative of more sex toy use, which might be more prevalent in high-income countries, is optimistically oriented and refers to extra-pleasurable masturbation due to more or less sophisticated technical aids. Such mass media information about sex toys has a normalizing and educational character. At the same time, it is clever public relations work on the part of the sex toy industry because specific brands and products (e.g., Womanizer.com and We-Vibe.com) have often been praised in media reports and, in some cases, press articles directly linked to online shopping sites.

This media narrative is often missing a critical examination of the commercialization of solo sex and of the sex products themselves, such as their functionality, sustainability, material quality, or pricing. While there are numerous blogs dedicated to the differentiated review of sex toys from users’ perspectives, only the marketing managers of the companies that report on sales successes have a say in media reports (Döring & Walter, 2020).

Empirically, we hardly know anything about pandemic-specific effects: By whom are sex toys increasingly bought, and how intensively are they being used during the COVID-19 pandemic? Which sexual scripts are acted out with these toys? What effects on solosexual satisfaction are experienced?

Finally, it should be noted that the narrative of more sex toy use refers to established toys such as dildos and vibrators and excludes sex dolls or sex robots, which are otherwise discussed as new trends (Döring, Mohseni, & Walter, in press; Döring & Pöschl, 2018). Whole-body toys such as robots and dolls, as well as conventional cuddly toys, may be particularly well suited for use in self-isolation when the need for tenderness, hugging, and cuddling is at stake and people want to calm and comfort themselves while falling asleep.

### More Pornography Use

During masturbation, people resort not only to toys but often also to pornography, currently mostly in the form of online pornography (Grubbs, Wright, Braden, Wilt, & Kraus, 2019). During the COVID-19 pandemic, this has increasingly been the case, at least that is what the narrative of more pornography use circulating in the media claims: “Corona makes porn purchases explode” was the headline of Germany’s largest daily newspaper (Döring & Walter, 2020).

Pornhub.com led a special public relations coup. This leading provider of online pornography made headlines worldwide at the beginning of March 2020 because it gave quarantined Italians free premium access to the platform for one month. The offer was so positively received that Pornhub immediately expanded it to Spain and France and eventually the whole world. Pornhub tweeted the following on March 24, 2020 (Pornhub, 2020b): “Stay home and help flatten the curve! Since COVID-19 continues to impact us all, Pornhub has decided to extend Free Pornhub Premium worldwide until April 23rd. So enjoy, stay home, and stay safe https://pornhub.com/stayhome #StayHomehub.”

Those who signed up for free also confirmed that they would stay away from social contacts and enjoy Pornhub premium videos in return. Pornhub jokingly changed its name to *StayHomehub*. For the Pornhub platform, which is repeatedly mentioned in the narrative of more pornography use, the COVID-19 crisis is likely to have resulted in a significant increase in the platform’s popularity. Pornhub’s approach was not at all original, as various companies have offered free premium access during the pandemic. In connection with porn, however, this marketing measure had a particularly high news value.

Pornography and its effects are a very controversial subject in both public and academic discourses. In the German-language media in the Spring of 2020, the narrative of more pornography use was accompanied by rather affirmative assessments: people staying at home and masturbating more often using pornography were suddenly seen less as a problematic risk behavior and more as a desirable SARS-CoV-2 prevention behavior and thus much more favorably regarded than usual. Professional assessments also go in this direction and do not see temporarily increased pornography consumption as a major problem but rather as constructive coping behavior to overcome pandemic-induced boredom and fear (Grubbs, 2020; Lehmiller, 2020) and to comply with contact bans (Grubbs, 2020):

For most users, pornography is probably just another distraction–one that might actually help “flatten the curve” by keeping people safely occupied and socially distanced. Combined with the fact that many people are isolating alone, pornography may provide a low-risk sexual outlet that does not cause people to risk their own safety or the safety of others.

In the international press, however, there are also articles that classify increased pornography consumption during the COVID-19 pandemic as a danger, mainly because it is expected to foster sexualized violence (Quek & Tyler, 2020; Schilling, 2020).

From a clinical point of view, it is predicted that in people who already have problems with their pornography consumption and self-regulation, these problems will increase under the conditions of the pandemic (Mestre-Bach et al., 2020). Hence, target-group-specific healthcare is necessary, for example, via online self-help forums (e.g., NoFap, Reboot Nation, or 12-step forums focusing on sex and love addiction) according to pornography addiction experts (Mestre-Bach et al., 2020).

Again, special attention should be paid to young people who may be consuming more pornography during the pandemic and at the same time have less direct contact with peers and sexual educators. They may need target-group-specific online sexual education during the pandemic. Moreover, positive effects are also possible, for example, in the form that the increased use of pornography may help in the exploration of one’s own sexual fantasies and desires, in self-validation and in open partner communication (Kohut, Fisher, & Campbell, 2017).

Instead of playing opportunities and risks against each other in a pro and con pornography discourse, according to the differential susceptibility to media effects model (DSMM; Valkenburg & Peter, 2013), we must identify the specific predictors that in individual cases can lead to predominantly positive, predominantly negative, ambivalent, or even no effects of pornography use.

### The Rise of Coronavirus Porn as a New Genre of Pornography

The media has reported not only about a general increase in pornography use but also about the rise of *coronavirus porn* as a new genre of pornography. In so-called coronavirus porn, the action takes place in a hospital or supposedly in the Chinese city of Wuhan, where COVID-19 originally broke out. The protagonists wear masks, gloves, and protective suits and interact with doctors and nurses, as described in detail by the magazine *VICE* (Cole, 2020). In fact, at the beginning of April 2020, more than 1000 videos were found on the pornography platform Pornhub.com under the search term “Corona” and more than 100 videos were found on the platform XHamster.com.

What has often been declared by the media to be an allegedly “extreme” or at least “strange” sex trend is actually quite normal: current events are reflected in people’s sexual fantasies and thus also become the subject of pornography. Coronavirus fantasies and porn probably have very different functions: fear defense, eroticization of the threat, curiosity about the bizarre, desire to cross borders, hopes of recovery, etc. (Lehmiller, 2020). Moreover, the motifs of coronavirus porn are partly connectable to existing fetishes and kinks (e.g., latex masks and gloves, clinic sex/white sex). While *Forbes Magazine* has emphasized the normality of eroticizing the novel coronavirus (Cookney, 2020), the *Independent* has criticized that an irresponsible pornography industry is capitalizing on the topic of coronavirus in a harmful way (Austin & Boyd, 2020).

Pornhub.com provided platform statistics that demonstrate increased interest in coronavirus porn in March 2020 (Pornhub, 2020a). These statistics were often quoted in the relevant media reports, which in turn may have helped to raise the popularity of the Pornhub brand. To date, there are no systematic content analyses of the new coronavirus pornography genre nor are there any empirical user studies on actual usage patterns and their possible effects.

## COVID-19 Effects on Sexual and Reproductive Health Issues in Vulnerable Groups

Both academic publications and the media point out that under the conditions of the COVID-19 pandemic, serious negative effects on sexual and reproductive health are to be predicted in the form of increased social and health inequalities. Particularly strong negative effects are expected among disadvantaged and neglected groups, including adolescents, those in humanitarian settings, people who identify as LGBTQ+, people experiencing gender-based violence, those living with HIV, incarcerated populations, individuals with disabilities, people of lower socioeconomic status, and people in low- and middle-income countries (Riley et al., 2020). As gender, class, and race are relevant factors, an intersectional perspective of sexuality-related COVID-19 effects is important (Hall et al., 2020). Some negative effects are classified as direct pandemic consequences (e.g., increasing sexualized domestic violence) and others are classified as indirect pandemic consequences (e.g., poorer medical care for pregnant women and newborns due to general overloading of the healthcare system).

### More Sexualized Domestic Violence

The prediction of the narrative of more sexualized domestic violence is that violence will escalate in domestic coronavirus isolation and that those affected find it even more difficult than usual to seek and receive help. The police, child protection associations, women’s shelters, and the media have issued warnings, published telephone hotline numbers and online contact points, and called for more room in women’s shelters (e.g., Bettinger-Lopez & Bro, 2020; Talmazan, Sirna, Ratto, & Ing, 2020). The WHO (2020b) has provided answers to central questions on violence against women in the COVID-19 pandemic. To provide care and protection for victims of sexualized domestic violence under pandemic conditions, it is also important to offer alternative violence reporting mechanisms and to avoid disruptions to the criminal justice system (Bettinger-Lopez & Bro, 2020): “Countries have shifted to virtual court hearings, facilitated online methods for obtaining protection orders, and communicated their intentions to continue to provide legal protection to survivors.”

The improved sensitization to the long-tabooed issue of sexualized domestic violence expressed in the media narrative of more sexualized domestic violence is very gratifying. At the same time, it is striking (and surprising in view of the extensive #MeToo debate on sexual assaults in educational and work contexts as well as in public) that the possible effects of the COVID-19 pandemic on sexualized violence in nondomestic contexts have hardly been discussed so far. Will more home offices, more home schooling, and more social distancing protect many children, adolescents, and adults—at least temporarily—from unwanted sexualized body contact in various abuse relationships (e.g., at school and at work) and from strangers (e.g., on the street and on public transportation)? Could coronavirus contact bans possibly make it easier for these individuals to get away from the perpetrator long enough to get help? What prevention and intervention services do we need to address these possible opportunities to end abusive relationships during the pandemic with its enforced contact bans?

At the same time, we must expect an increase in sexualized violence (mostly against women and children) in digital contexts due to the increased use of online communication and counteract it with appropriate prevention and intervention measures (Parks et al., 2020; UN News, 2020).

### More Barriers to Family Planning and Abortion

The media narrative of more barriers to family planning and abortion indicates that under the conditions of the COVID-19 pandemic with curfews and limited medical care, there is sometimes insufficient access to contraception and abortion. This applies to high-income countries such as Germany, where during the COVID-19 pandemic, access to mandatory offline abortion counseling was difficult. To alleviate this problem, for the first time, online counseling has been allowed in this field. However, the demand of pro-choice activists to enable telemedical-accompanied medical abortion at home, which is already being practiced in other countries and has also been positively evaluated scientifically (Raymond et al., 2019), has not been met politically in Germany (Döring & Walter, 2020).

In middle- and low-income countries, the shortcomings in the supply of contraceptives and in access to abortion are even greater. A model calculation for 132 low- and middle-income countries showed that a deterioration in the two areas mentioned by only 10% within 1 year will lead to more than 15 million additional unintended pregnancies and more than 3 million additional unsafe abortions, with more than 1000 additional maternal deaths (Riley et al., 2020). From the experience with the Ebola epidemic in West Africa (Bietsch, Williamson, & Reeves, 2020; Camara et al., 2017; Sochas, Channon, & Nam, 2017), it can be concluded that the reproductive healthcare problems caused by the COVID-19 pandemic in low- and middle-income countries will be serious and long term.

The professional community therefore calls for sexual and reproductive healthcare to be defined as essential, for support programs to be set up and for care facilities and care measures to be adapted (e.g., distribution of contraceptives without a prescription and telemedically assisted medical abortion; Hall et al., 2020; Riley et al., 2020).

### More Health Risks During Pregnancy and Birth

The existential experiences surrounding pregnancy and birth are generally accompanied by fears and uncertainties. Under the conditions of a pandemic, these are even more intense: the risk of infection, supply bottlenecks in clinics, and the question of whether a personal companion may be taken into the delivery room although contact restrictions are common worries. The mass media reports in high-income countries have displayed such worries and fears, reporting on anxiety and desperation among pregnant women. “Strained hospitals and isolation: How coronavirus made giving birth even harder” was the title of an article in the British *Guardian* (Graves, 2020). However, the press has also reported on new help services such as Facebook groups by and for pregnant women, online birth preparation courses, and virtual midwifery support (Döring & Walter, 2020).

In middle- and low-income countries, the provision of essential pregnancy-related and newborn care has become even more problematic during the COVID-19 pandemic. According to model calculations, a decline in care by only 10% would lead to more than 4 million additional serious complications for mothers and children and to almost 200,000 additional deaths of mothers and newborns (Riley et al., 2020). Here, too, it is therefore necessary to counteract the feared and presumably already occurring supply bottlenecks with rapid and sustainable measures (Hall et al., 2020). There are additional risks of SARS-CoV-2 infection for pregnant women and newborns (Rasmussen et al., 2020); however, the first case studies found no evidence for vertical transmission in late pregnancy (Chen et al., 2020). For HIV-uninfected pregnant and breastfeeding women at risk of HIV acquisition, continued PrEP provision and HIV risk reduction counseling are explicitly recommended by experts from South Africa, even if visits to possibly crowded healthcare facilities may increase their risk of contracting SARS-CoV-2 (Joseph Davey et al., 2020).

### More Health Risks and Economic Hardships in Sex Work

A relatively large number of media reports point to existence-threatening situations for prostitutes and other sex workers resulting from brothel closures and bans on body-related services, which, due to the lack of reserves in most cases, lead to acute poverty and homelessness (Döring & Walter, 2020). Interestingly, the media reports presenting the narrative of more economic hardships in sex work often not only speak “about” sex workers but actually with them: sex work organizations and individual sex workers have their say in detail. In view of the polarized debates on prostitution, which are often conducted without the people concerned and morally condemn the offering and/or demand of sexual services (see Benoit, Smith, Jansson, Healey, & Magnuson, 2019), it is remarkable that in relation to the COVID-19 impact, the human and labor rights of sex workers have often been so visibly placed on the media agenda (e.g., in Germany) (Döring & Walter, 2020). However, the coronavirus pandemic is also used by the anti-prostitution movement to campaign for further criminalization of prostitution. The novel coronavirus has already been called the “Abolitionist virus” because it prevents men from paying for sex (Smith, 2020). UNAIDS (2020) has explicitly demanded the protection of the health and rights of sex workers during the COVID-19 pandemic and the involvement of sex workers in emergency public health planning groups. Meanwhile, sex work organizations worldwide have demonstrated resourcefulness in responding to the pandemic by engaging in mutual help, building emergency hardship funds, and organizing undisrupted sexual health care (Kimani et al., 2020; Smith, 2020).

In addition to sex workers offering in-person services for clients, pornography performers have also been severely affected by COVID-19 as porn production studios have closed. To stay in business, some sex workers and pornography performers now market phone sex and online sex or sell solo sex recordings (Lehmiller, 2020) and additional products (e.g., worn underwear and other fetish items). The international survey on the perceptions of sex workers and sex work organizations of COVID-19 effects (https://www.nswp.org/news/nswp-launch-covid-19-impact-survey), run by the Global NSWP, will provide the first empirical data on those issues. According to media reports, the existence of small sex shops without online distribution is also threatened.

### More Disadvantages for LGBTQ+-Identifying Populations

In two open letters, more than 100 LGBTQ + organizations in the U.S. have pointed out that LGBTQ + communities are being severely affected by the negative consequences of the COVID-19 pandemic (National LGBT Cancer Network, 2020; Whitman-Walker Health et al., 2020). This is attributed to the fact that LGBTQ + communities are vulnerable due to increased rates of smoking, cancer, and HIV and thus belong to COVID-19 risk groups. Furthermore, the open letters stress that LGBTQ + people experience discrimination, unwelcoming attitudes, and lack of understanding from providers and staff in many healthcare settings and, as a result, many are reluctant to seek medical care. LGBTQ + people are disproportionately low income or live in poverty according to the open letters. Consequently, there is a demand to investigate the support needs of LGBTQ + populations more closely and to provide nondiscriminatory healthcare and economic support during the pandemic.

Scientific contributions address, among other things, the importance of HIV, PEP, and PrEP care during the COVID-19 pandemic (Alpalhão & Filipe, 2020; Junejo et al., 2020; Sun et al., 2020). They point to the discrimination of transgender and gender nonconforming (TGNC) people by national COVID-19 measures (e.g., in Peru) (Perez-Brumer & Silva-Santisteban, 2020) and explain the multiple (mental) health challenges of TGNC individuals when their gender-affirming treatment is deferred during COVID-19 (van der Miesen, Raaijmakers, & van de Grift, 2020). Another important research topic is the new COVID-19 stigma and how to combat it (Logie & Turan, 2020). Press reports have indicated that coronavirus is also discussed in xenophobic and racist frames (“China virus” or “Wuhan virus”) and in homophobic and transphobic frames (“Homo virus”; Browning, 2020). Coronavirus tracking measures can be problematic if they contribute to stigmatization of LGBTQ + persons, such as in South Korea (Borowiec, 2020).

## COVID-19 Effects on Sexual and Reproductive Healthcare and Sexual Education

Looking at the sexuality-related COVID-19 effects from the perspective of sexual healthcare provision, the following four aspects are particularly relevant, as mentioned in some of experts’ comments (e.g., Hall et al., 2020; Joseph Davey et al., 2020; Mestre-Bach et al., 2020; National LGBT Cancer Network, 2020; Rasmussen et al., 2020; Riley et al., 2020; Thorne et al., 2020; UNAIDS, 2020; Whitman-Walker Health et al., 2020). They are not very visible in media reporting, though.

1. Sexual and reproductive healthcare that requires personal consultation and treatment (e.g., sexual health-related surgeries, safe delivery, contraception and abortion services) must be guaranteed in times of pandemics and even expanded as needed (e.g., because of possibly increasing pregnancy rates), while infection protection measures are taken. Here, especially in low- and middle-income countries, support programs and infrastructure measures are necessary.
2. Wherever possible, sexual and reproductive healthcare services and sexual education should be transferred from offline settings to infection-proof online settings accessible during curfews and contact bans in times of pandemics. Corresponding online platforms, mobile apps, and virtual therapy and counseling services must be developed, implemented, and evaluated. In its recent position statement, the European Society for Sexual Medicine (ESSM) emphasized the great potential of *e*-*sexual health* (Kirana et al., 2020); however, using telemedicine in sexual health comes with diverse challenges (Shindel & Rowen, 2020). Nonetheless, good practice examples are already available (e.g., for the provision of comprehensive HIV PrEP service via telehealth for adolescent MSM and TGW in Brazil: Dourado et al., 2020; for the prevention of consumption of child sexual abuse material via online cognitive behavioral therapy: Parks et al., 2020).
3. In addition to the development of new digital delivery formats, sexual and reproductive healthcare services and sexual education are required to incorporate pandemic-specific topics such as coping with domestic isolation for couples experiencing increased domestic estrangement or conflicts, coping with domestic isolation for singles experiencing increased loneliness, and dealing with technology-mediated sexual interactions and relationships among adolescents, adults, and seniors. Appropriate target-group-specific content, which is rights-based, inclusive, pleasure-friendly, and simultaneously focuses on risk reduction, must be developed, implemented, and evaluated. Some authors suggest that pandemic-related health interventions should include positive sexual health and even promote safe and consensual sexual activity in sexually inactive people to mitigate some of the detrimental consequences of self-isolation and social distancing (Jacob et al., 2020). The assumed reduction in the number of sex partners and in sexual risk taking during the pandemic creates a good opportunity for STI and HIV prevention to find undiagnosed people (Junejo et al., 2020). Hence, pandemic-specific social media campaigns that encourage testing are recommended and already implemented in the UK (e.g., https://www.testnowstophiv.com/), using claims like “Been going solo recently? Time to test. Lockdown has broken the HIV chain.”
4. To meet these new challenges (e.g., the implementation of infection control in face-to-face service settings, development of new digital delivery formats, and the development of new target-group-focused pandemic-specific content and services), professionals in the field of sexual and reproductive healthcare and sexual education, for their part, need appropriate (virtual) training courses, concepts, tools, and economic support. For example, sexual education professionals and institutions are currently facing existential problems, even in high-income countries, because they have been unable to hold their usual face-to-face events, seminars, and workshops for months.

An important prerequisite for appropriate measures is the recognition of sexual and reproductive healthcare and sexual education as being essential in times of a pandemic.

## Discussion and Outlook

This overview of current media narratives and scientific observations and predictions on sexuality-related COVID-19 effects shows how many different aspects of sexual behavior and sexual and reproductive health can be affected. Looking at the public and professional discourse, it is gratifying that questions of sexual and reproductive health and rights have moved so quickly onto the agenda. It is also gratifying that underserved groups and their demands are so clearly visible in the discussion and that their voices are being heard. It is also remarkable that technology-mediated sexual interactions and masturbation with pornography and toys under conditions of a pandemic with contact restrictions have suddenly been normalized to such a degree that they are even officially recommended by the media and health authorities as health prevention behaviors.

As with any change, we probably tend to accentuate either positive or negative effects, neglecting the possibility of only small effect sizes and the possibility of ambivalent effects. This can be assumed, for example, for the pandemic effects on partnered sex and solo sex: since relationship sex and masturbation are in part strongly habitualized, the pandemic can have only a temporary, limited, and often ambivalent impact. Other consequences of the pandemic, however, appear to be clearly negative, serious, and long term, such as the lack of access to family planning and abortion, poorer care during pregnancy and birth, or the rise of a racist and homophobic coronavirus stigma.

Overall, it is important to empirically test the common media narratives and research hypotheses that have been put forward and to develop intervention measures that will help to better protect and strengthen sexual and reproductive health in future epidemics and pandemics. It is particularly important to keep in mind the feared worsening of inequalities in sexual and reproductive healthcare at national and international levels and to initiate rapid and sustainable countermeasures. Questions of resilience and coping in connection with sexual and reproductive health under the conditions of a pandemic are also worth investigating, both on the side of sexual healthcare providers and on that of their patients and clients.

The question of a possible long-term cultural change is also relatively open at this point in time. Will technology-mediated forms of sex remain more common and more recognized after they have been used during the pandemic contact bans in broader sections of the population and were recognized as preventive behaviors? Will the rules of social distancing be more firmly anchored in everyday life in the long term, or will a return to the former status quo occur relatively soon?

This commentary provides a first overview of media narratives and research hypotheses on the sexuality-related effects of the COVID-19 pandemic. The main limitations of the present commentary are that only German and English language media contributions and academic papers were included and that the number of empirical studies in this field is still very small. The planned special issue of *Archives of Sexual Behavior* on the impact of COVID-19 on sexual health and behavior will help to close the diverse research gaps (Scott-Sheldon, Mark, Balzarini, & Welling, 2020).
